# Neoadjuvant Concurrent Chemoradiation for Curative Treatment of Penile Squamous Cell Carcinoma

**DOI:** 10.1155/2014/479376

**Published:** 2014-10-07

**Authors:** Arpit Chhabra, David Schwartz, Andrea Leaf, Nikolaos Karanikolas, Jeffrey P. Weiss, David Schreiber

**Affiliations:** ^1^Department of Veterans Affairs, New York Harbor Healthcare System, 800 Poly Place, Suite 114A, Brooklyn, NY 11209, USA; ^2^Downstate Medical Center, State University of New York, Brooklyn, NY 11203, USA

## Abstract

*Introduction*. Penile cancer is a rare malignancy often treated with neoadjuvant chemotherapy followed by surgery. However, the utility of neoadjuvant chemoradiation, particularly when the tumor is resistant to chemotherapy alone, has not been established. In this study, we report a case of pT3cN3M0 penile squamous cell carcinoma with progression of nodal disease on chemotherapy, which was cured with use of neoadjuvant concurrent chemoradiation.* Case Report*. A 65-year-old male presented with a fixed left inguinal lymph node with associated firmness of the penile glans. Biopsies of both sites revealed evidence of squamous cell carcinoma. The patient underwent partial penectomy for the primary lesion and began neoadjuvant chemotherapy to reduce the size of the unresectable left inguinal node. However, he displayed disease progression in the left inguinal node. As such, we attempted concurrent chemoradiation therapy with regression of his nodal disease. The patient was able to undergo left inguinal node dissection and has no evidence of disease 18 months since his initial surgery.* Conclusion*. The use of neoadjuvant chemoradiation for bulky cN2-3 disease seems appropriate in the setting of progressive disease. Further studies are necessary to assess the utility of concurrent chemoradiation both in the neoadjuvant and salvage setting.

## 1. Introduction

Penile carcinoma represents a rare disease entity in the developed world, accounting for less than 1% of cancers in men in the United States. The incidence, however, displays a significant geographic variation with penile cancers occurring more frequently in Asia, Africa, and South America [1]. Surgical resection remains an integral treatment modality for penile carcinoma in addressing both the primary lesion and lymph node metastases. However, at least 8% of patients can present with bulky and/or fixed nodal disease (N2-N3) [2].

Historically, such patients were deemed unresectable and provided palliative radiation and chemotherapy. In 2007, however, Leijte et al. conducted an institutional review of 20 patients who received neoadjuvant chemotherapy, with the majority receiving bleomycin based therapy, in an attempt to downstage unresectable regional and locally advanced disease [3]. Of the 20 patients, 17 presented with fixed lymph node metastases (Tx, N3). 12 clinical complete and partial responses were achieved using neoadjuvant chemotherapy, with a statistically significant survival difference between responders and nonresponders.

Given this study's results and the analyses that followed, neoadjuvant chemotherapy became incorporated in the paradigm of treating bulky and fixed lymphadenopathy prior to inguinal lymph node dissection. Unfortunately, the survival advantage of neoadjuvant therapy is only present for chemotherapy responders. In this current report, we therefore highlight the benefit of using neoadjuvant concurrent chemoradiation for patients who are nonresponders to neoadjuvant chemotherapy alone.

## 2. Case Report

**Figure 1:**
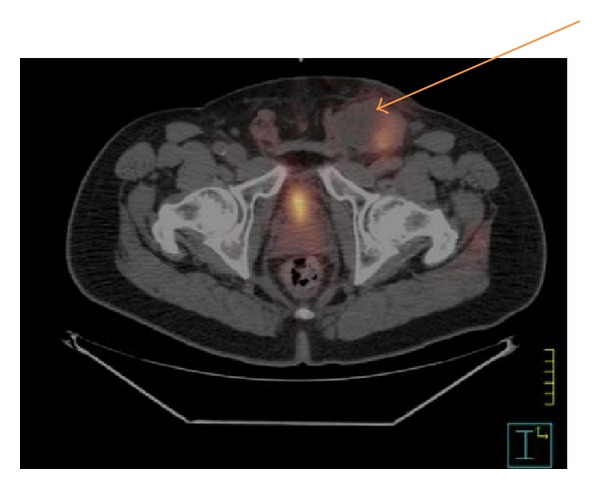
Initial staging PET-CT revealed SUV uptake at the base of the penis (not shown) as well as SUV uptake in a left inguinal lymph node (shown above).

A 65-year-old African-American male presented with recurrent bouts of dysuria for four months, which were unsuccessfully treated with multiple courses of antibiotics. The patient subsequently developed hematuria, for which he was scheduled to undergo cystoscopic evaluation. In the interim he developed a large 5 cm fixed left inguinal lymph node. Physical examination also revealed a firm indurated glans with no other relevant findings. Cystoscopy was eventually completed, revealing an extrinsic lesion invading into the urethra at the fossa navicularis, which was biopsied, as was the left inguinal lesion. Pathology of both samples confirmed poorly differentiated squamous cell carcinoma. Staging PET-CT revealed increased glucose uptake at the base of the penis (SUV 5.6) as well as hypermetabolic uptake in a left inguinal lymph node (SUV 8.0) with no other pieces of evidence of metastatic foci (Figure 1). The patient was, therefore, completely staged as pT3cN3M0 (AJCC Staging 7th edition).

He subsequently underwent a partial penectomy with attempted bilateral inguinal dissection; pathology revealed moderately differentiated, invasive squamous cell carcinoma involving the urethra. Surgical margins were negative (pT3). However, left inguinal dissection was omitted, as the nodes were fixed and therefore unresectable. As such, the patient was considered for neoadjuvant chemotherapy with 4 cycles of paclitaxel, ifosfamide, and cisplatin (TIP). However, repeat imaging with CT after 2 cycles of neoadjuvant TIP revealed that the left inguinal mass had increased in size, measuring 6.9 cm in largest diameter. Given the progressive disease on imaging, the patient was referred for consideration of concurrent chemoradiation therapy.

He began chemoradiation therapy receiving 4500 cGy in 180 cGy/fx using intensity modulated radiation therapy to cover the bilateral inguinal nodes, pelvic lymph nodes up to L5-S1, and penile base. Since his disease had progressed on platinum based therapy, he was offered concomitant Capecitabine 500 mg BID as an alternative. The patient tolerated his chemotherapy and radiation treatments well. He was initially planned to receive a projected total dose of 6660–7020 cGy. However, his radiation treatments were suspended at 4500 cGy due to significant regression of the left inguinal node, which had also converted from fixed to mobile. He was therefore reassessed by surgery and was able to undergo a formal left inguinal lymph node dissection. Pathology of the dissection revealed small foci of metastatic poorly differentiated squamous cell carcinoma embedded in fibrous connective tissue. The patient did not undergo right inguinal or pelvic lymph node dissection given prior radiation field coverage.

**Figure 2:**
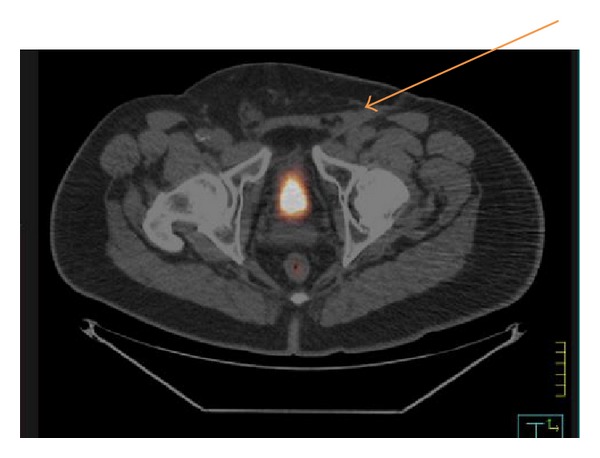
Most recent PET-CT revealing no evidence of disease uptake.

Two months after completion of the most recent surgery, PET-CT imaging was completed revealing hypermetabolic soft tissue opacity in the region of the resected left inguinal node, which could not be further characterized. Despite recommendation of additional radiation therapy given the uptake in the left groin, the patient was lost to followup for 4 months. Upon returning, he underwent repeat CT A/P which revealed an abnormal left inguinal soft tissue mass measuring 3.7 cm in greatest diameter at the site of his prior adenopathy, albeit with decreased size radiographically. Upon radiological review, this was felt to be consistent with postoperative changes rather than recurrent disease. Therefore, the patient continued on close surveillance and was seen in followup 4 months later, at which time PET-CT was repeated, revealing no abnormal SUV uptake (Figure 2). The patient remains in active followup and shows no evidence of disease both clinically and radiographically at 18 months since completing his initial surgery.

## 3. Discussion

Surgical resection remains the primary treatment for penile squamous cell carcinoma, achieving good local control rates at the primary site with partial or total penectomy [4]. However, it is the involvement of regional lymph nodes that serves as the single most important prognostic factor for penile squamous cell carcinoma [5]. Therefore, management of lymph nodes needs to be well delineated in order to achieve long-term cure. Patients without palpable inguinal lymph nodes are generally referred for lymph node evaluation depending on the risk factors associated with their primary tumor, whereas all patients with palpable inguinal lymph nodes necessitate evaluation with a fine needle aspiration to diagnose metastatic involvement. Involved nodes should be followed subsequently by an inguinal lymph node dissection, which can be curative in situations of limited lymph node metastases [6]. However, surgery alone achieves suboptimal outcomes in patients with more extensive lymph node metastases with 5-year OS and DFS rates as low as 10–20% for pN2-N3 disease [7]. These patients, therefore, require more aggressive multimodal therapies. Chemotherapy has been the best studied in this respect both in the adjuvant and in the neoadjuvant settings in an attempt to improve outcomes for pN2-N3 patients.

The European Association of Urology and NCCN guidelines both state that adjuvant chemotherapy can be considered for those with pN2-N3. These recommendations are based predominantly on a retrospective study by Pizzocaro et al., which compared 12 patients with surgically resected nodes who received vincristine/methotrexate/bleomycin adjuvant chemotherapy. The outcomes of this cohort were compared with historic controls and revealed a 5-year survival rate of 82% with the addition of adjuvant chemotherapy compared with 37% for the historical control group [8, 9]. Other than the analyses of Pizzocaro et al., only a few other small case control series comment on adjuvant chemotherapy. As such, there is not sufficient evidence to make definite conclusions regarding the use of adjuvant chemotherapy.

Neoadjuvant chemotherapy on the other hand has shown promising results in recent literature especially in the setting of cN2-N3 patients with large, multiple, and/or immobile nodes. Pagliaro et al. evaluated outcomes of TxcN2-N3M0 patients having received 4 cycles of neoadjuvant chemotherapy with paclitaxol/ifosfamide/cisplatin (TIP) followed by surgical inguinal and pelvic lymph node dissection for patients fit enough for surgery or who achieved a radiographic complete response to chemotherapy [10]. Of the 30 patients receiving neoadjuvant chemotherapy, 15 patients achieved either a clinical complete or a partial response, 9 patients showed stable disease, and the remaining 6 patients had progression of disease. Twenty-three patients completed all four cycles of chemotherapy, of whom 22 underwent surgery. The long-term overall survival for this cohort was 33% at a median followup of 34 months, with time to progression and overall survival significantly associated with response to chemotherapy (*P* < 0.001). Based on the findings of this analysis, the NCCN strongly recommends the use of neoadjuvant chemotherapy, followed subsequently by surgery for responding or stable nodal disease. Unfortunately, this leaves out patients who are neoadjuvant chemotherapy nonresponders, including the one presented in this case report, for whom management is not as clear. This raises the possibility of using radiation therapy as a neoadjuvant after a nonresponse has been appreciated.

Adjuvant radiation therapy for cN+M0 metastatic penile carcinoma has been studied only sparsely. Based on extrapolation of its benefit in other sites including the vulvar one, the use of adjuvant radiotherapy has been considered in the setting of a surgical lymph node dissection with multiple positive inguinal nodes or evidence of extracapsular extension on pathology. In one of the few retrospective reviews on this topic, Chen et al. showed that the addition of adjuvant radiation therapy at a dose of 54 Gy in patients with pN+ disease reduced the regional failure rate to 11% from 60% without radiation therapy (*P* = 0.057) [11]. Unfortunately, given the small sample size of this study and the dearth of other studies, further evaluation regarding the use of adjuvant radiation therapy is necessary.

Based on the rarity of penile squamous cell cancer, little also exists with regard to the use of radiation therapy in the neoadjuvant setting for unresectable cN+ patients. In low volume cN+ patients, preoperative radiation therapy has been shown to be highly effective in sterilizing nodes and reducing nodal recurrences [12]. Our case report is one of the few to establish the feasibility of neoadjuvant concurrent chemoradiation in patients with cN2-N3 disease including bulky or fixed inguinal lymph nodes. Our patient received neoadjuvant chemoradiation in the setting of progressive disease after neoadjuvant chemotherapy, though the use of neoadjuvant chemoradiation need not be limited to this scenario. One may also consider using neoadjuvant chemoradiation as an initial treatment modality for bulky or fixed cN2-N3 disease in hopes of achieving a curative surgical dissection.
